# Low‐dose X‐ray radiation induces an adaptive response: A potential countermeasure to galactic cosmic radiation exposure

**DOI:** 10.1113/EP091350

**Published:** 2024-01-05

**Authors:** Siena Edwards, Jessica Adams, Anastasia Tchernikov, John G. Edwards

**Affiliations:** ^1^ AresVallis LLC Valhalla New York USA

**Keywords:** cardiomyopathy, DNA damage, galactic cosmic radiation, space flight countermeasures

## Abstract

Space exploration involves many dangers including galactic cosmic radiation (GCR). This class of radiation includes high‐energy protons and heavy ionizing ions. NASA has defined GCR as a carcinogenic risk for long‐duration space missions. To date, no clear strategy has been developed to counter chronic GCR exposure. We hypothesize that preconditioning cells with low levels of radiation will be protective from subsequent higher radiation exposures. H9C2 cells were pretreated with 0.1 to 1.0 Gy X‐rays. The challenge radiation exposure consisted of either 8 Gy X‐rays or 75 cGy of GCR, using a five‐ion GCRsim protocol. A cell doubling time assay was used to determine cell viability. An 8 Gy X‐ray challenge alone significantly (*P* < 0.05) increased cell doubling time compared to the no‐radiation control group. Low‐dose radiation pre‐treatment ameliorated the 8 Gy X‐ray‐induced increases in cell doubling time. A 75 cGy GCR challenge alone significantly increased cell doubling time compared to the no‐radiation group. Following the 75 cGy challenge, only the 0.5 and 1.0 Gy pre‐treatment ameliorated the 75 cGy‐induced increases in cell doubling time. DNA damage or pathological oxidant stress will delay replicative functions and increase cell doubling time. Our results suggested that pretreatment with low‐dose X‐rays induced an adaptive response which offered a small but significant protection against a following higher radiation challenge. Although perhaps not a practical countermeasure, these findings may serve to offer insight into cell signalling pathways activated in response to low‐dose irradiation and targeted for countermeasure development.

## Abstract (highlights)

- **What is the central question of this study?**
  Space travel increases galactic cosmic ray (GCR) exposure, which produces more complex DNA damage requiring longer repair periods with more mis‐repairs: does a low‐dose low linear energy transfer (LET) pretreatment stimulate a hormetic response that is protective from later high‐LET GCR events?
- **What is the main finding and its importance?**
  A low‐dose low‐LET event induced a significant adaptive response that restored cell viability in response to GCR. Identifying the critical pathways of the adaptive response may identify targets for countermeasure development.

## 1 INTRODUCTION

Travel beyond Earth's atmosphere has several distinct hazards. Significant among them are galactic cosmic radiation (GCRs) and solar energetic particles (SEPs), which are energetically charged particles consisting of protons and heavier ions including ^56^Fe, ^28^Si and ^16^O (McLaughlin et al., 2017). GCRs and SEPs are classified as high linear energy transfer (LET) events as compared to X‐rays, which are low‐LET events. There are significant biological differences in the response to low‐ or high‐LET events.

GCR exposure is elevated in low Earth orbit (LEO) with flight crews aboard the International Space Station receiving about 0.4 mSv/day (Cucinotta et al., 2008). Beyond LEO during the transit to Mars, the expected fluency is estimated to be 1.8 mSv/day and while on the surface of Mars about 0.72 mSv/day (Cucinotta, 2015). Although the fluency of exposure is very low, the hazards of chronic exposure are in part evidenced by the study of airline pilots who demonstrate a small but significant increase in cancer incidence (Miura et al., 2019; Sanlorenzo et al., 2015).

The induction of an adaptive response to mild stress that is protective against a subsequent greater challenge is referred to as a hormetic effect. It has been observed across diverse stresses including myocardial ischaemia, chemical carcinogenesis, thermal stress and radiation exposure (Demple & Halbrook, 1983; Olivieri et al., 1984; Schwarz et al., 1997). The protective effects of low‐dose ionizing radiation have been reported across species and one report indicated that low‐dose gamma radiation extended the lifespan of mice (Caratero et al., 1998). Although previous studies using low‐LET radiation have suggested that pre‐conditioning may induced an adaptive response, the impact of high‐LET events on cells is not clear. To that end, we have examined if preconditioning cells with a low dose of X‐ray radiation may induce protection from a subsequent high‐LET GCR exposure.

## 2 METHODS

### 2.1 Cell culture

H9c2 myoblasts (American Type Culture Collection (ATCC), Manassas, VA, USA; CRL‐1446) were maintained according to ATCC recommendations (Dulbecco's modified Eagle's medium (DMEM) + 10% fetal bovine serum (FBS)).

### 2.2 X‐ray radiation

H9C2 cells maintained in culture were pretreated with a low dose of 0.1, 0.5 or 1.0 Gy X‐rays (1.1 Gy/min; 160 kV) using a Rad Source Technologies (Buford, GA, USA) RS 2000 Biological Irradiator. For the X‐ray challenge dose, cells were exposed to 8 Gy (1.1 Gy/min; 160 kV). Non‐irradiated control cells were maintained in the room shielded from the cells being irradiated.

### 2.3 Galactic cosmic radiation

Twenty‐four hours prior to the pretreatment dose, the cells were split to a 40% confluency into T75 or T25 flasks. The GCR challenge dose was performed 48 h later in the NASA Space Radiation Laboratory (NSRL) at Brookhaven National Laboratories (Upton, NY, USA). Cells underwent a single exposure of 75 cGy, following the five ion GCRsim configuration. Table 1 lists the beam order, beam energy and exposure dose for each ion species in the GCRsim protocol. Non‐irradiated control cells were maintained at the NSRL in a room separate from the cells undergoing GCR exposure. Following radiation treatment, GCR‐treated and control cells were returned to the incubator, and following deactivation, the medium was replaced with fresh medium.

**TABLE 1:** 75 cGy 5‐ion GCRsim protocol.

Ion species	Beam energy (MeV/*n*)	Delivered dose (cGy)
Proton	1000	26.25
Silicon	600	0.75
Helium	250	13.50
Oxygen	350	4.50
Iron	600	0.75
Proton	250	29.25

### 2.4 Cellular function tests

Several different protocols were used to evaluate cellular function. In brief, cells were split onto 96‐ or 24‐well test plates 2 days following the GCRsim challenge and maintained for 7 days or as indicated. Cell viability was determined using 0.5 mg/mL methyl thiazolyl tetrazolium (MTT; M5655, Sigma‐Aldrich, St Louis, MO, USA). In brief, 100 μL MTT dissolved in clear DMEM was put on cells for 4 h at 37°C. The medium was removed and replaced with 75 μL of acidified isopropanol. Absorbance was read using a Tecan (Baldwin Park, CA, USA) plate reader at 560 nm (690 nm: reference). Cytosolic superoxide levels were determined using dihydroethidium (DHE) fluorescence. DHE was added to a final concentration of 40 μM and cells were incubated in the dark for 45 min at 37°C. The cells were washed twice with phosphate‐buffered saline (PBS) before the plate was read on a Tecan plate reader (excitation at 498 nm; emission at 583 nm) (Zhao et al., 2003). Mitochondrial superoxide levels were determined using MitoSox fluorescence as described previously (Hicks et al., 2013). TMRE was used to measure mitochondrial membrane potential. In brief, tetramethylrhodamine ethyl ester perchlorate (TMRE, Sigma 87917) was added to a final concentration of 5 μM and the cells were incubated in the dark for 60 min at 37°C. The fluorescence was determined on a Tecan plate reader (excitation 530 nm, emission 580 nm).

Cell doubling time was adapted from the protocol of (Wolfs et al., 2013). All reagents for flow cytometry analysis, including cation‐free PBS, were filtered using a 0.45 μm filter to remove particulates. Cells were trypsinized and counted using the (Guava flow cytometer, Millipore, Billerica, MA) excluding cells less than 2μ. All cell transfers were made using a wide‐bore pipet tip. 10**^5^** cells were transferred into wells of a 24‐well test plate. Cells from each well were counted after 48–72 h using the Guava flow cytometer excluding cells less than 2 μm. For the 24‐well plates, cells were trypsinized using 100 μL 0.5 mM EDTA for 30 s followed by 200 μL 0.25% trypsin/2.21 mM EDTA for 60 s, before 600 μL FBS was added. Cells were then transferred to a microtube for counting.

### 2.5 Statistical analysis

The values shown are means and SD. Statistical analysis was done using one‐way ANOVA, with *post hoc* analysis using the Holm–Šidák method (NCSS, Kaysville, UT, USA or GraphPad Software, Boston, MA, USA). Unless otherwise indicated, statistical significance was set at *P* < 0.05.

## 3 RESULTS

**FIGURE 1:**
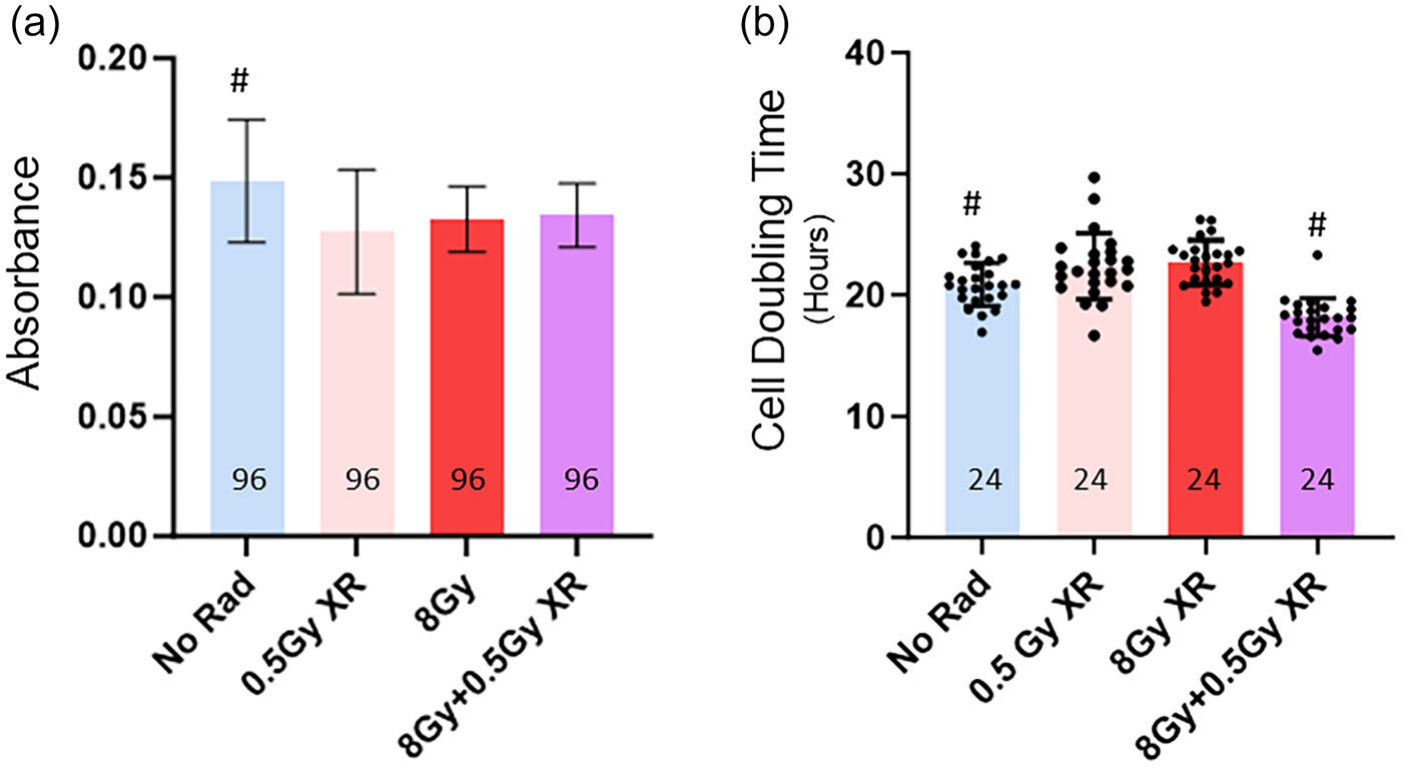
Low‐LET X‐ray exposure altered cellular doubling time. H9c2 cells were exposed to combinations of low‐dose and/or challenge X‐ray irradiation. (a) MTT measure of cell viability. (b) Cell doubling time analysis. Cells were exposed to 0.5 Gy X‐rays and where indicated this was followed 2 h later by an 8 Gy challenge dose. Cells were maintained and analysed 6 days post‐challenge. Values shown are means ± SD, with sample size indicated within each bar; #*P* < 0.05 compared to 8 Gy alone; detailed *post hoc* analysis is shown in Tables 2 and 3.

The cell doubling time protocol measures time required for mitosis, which DNA damage or pathological oxidant stress will delay. As such, it is a more physiological estimator for a cell's viability. H9c2 cells were exposed to low‐dose 0.5 Gy X‐rays 2 h before an 8 Gy X‐ray challenge dose. Seventy‐two hours following the challenge dose, cell viability was significantly depressed in the separate 0.5 Gy and 8 Gy X‐ray treatments (Figure 1a; Table 2) as compared to non‐irradiated control cells. A cell doubling time assay was initiated 72 h post‐challenge. The 8 Gy X‐ray challenge dose significantly increased the cell doubling time in comparison to non‐irradiated cells (Figure 1b). Low‐dose X‐ray pretreatment restored the cells’ replicative ability, as the cell doubling times (8 Gy + 0.5 Gy) were significantly lower compared to the 8 Gy challenge dose alone and not different from the non‐irradiated cells (Table 3).

**TABLE 2:** *P*‐values of direct comparisons for Figure 1a (MTT assay).

	vs no radiation	vs 8 Gy X‐rays
No radiation		<0.0001
0.5 Gy X‐rays	<0.0001	0.1317
8.0 Gy X‐rays	<0.0001	
8 Gy + 0.5 Gy X‐rays	<0.0001	0.6251

**TABLE 3:** *P*‐values of direct comparisons for Figure 1b (cell doubling time).

	vs no radiation	vs 8 Gy X‐rays
No radiation		0.0076
0.5 Gy X‐rays	0.0121	0.9152
8.0 Gy X‐rays	0.0054	
8 Gy + 0.5 Gy X‐rays	<0.0001	<0.0001

**FIGURE 2:**
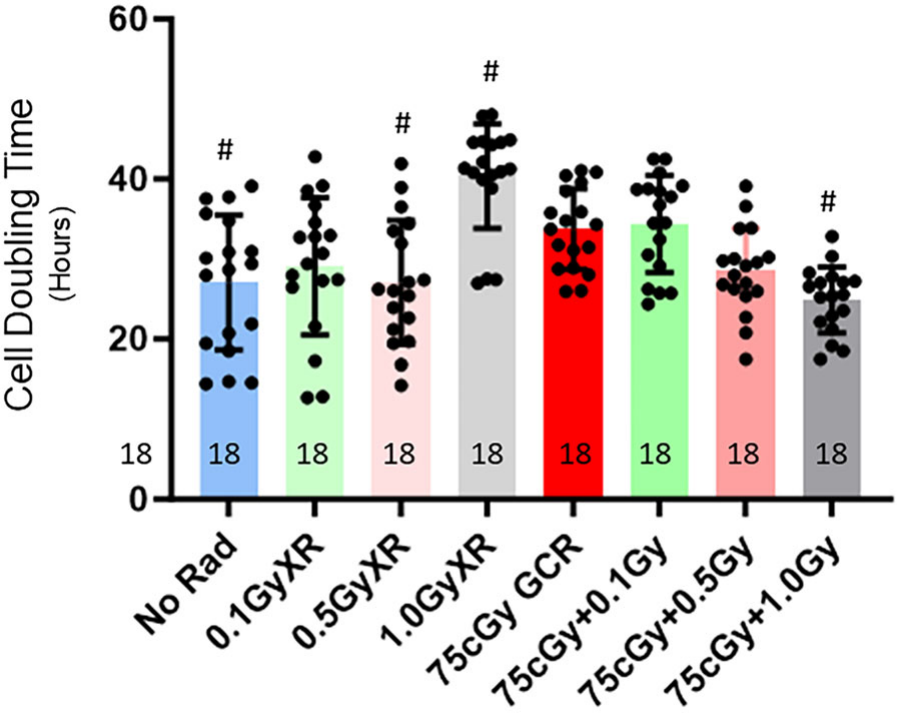
High‐LET radiation altered cell doubling time. H9c2 cells were exposed to low dose X‐rays and/or 75 cGy GCR irradiation. Cells were exposed to low dose X‐rays, and where indicated this was followed 3 days later by the 75 cGy GCR challenge dose. After 7 days post‐challenge, cells were transferred to test plates and counted 48–72 h post‐plating. Values shown are means ± SD, with sample size indicated within each bar; # *P* < 0.05 compared to 75 cGy alone; detailed *post hoc* analysis is shown in Table 4.

The GCR experiments used a different time frame due to operational parameters. Cells were exposed to low dose 0.1–1.0 Gy X‐rays 72 h before a 75 cGy GCR challenge dose. Cells were analysed 1 week post‐GCR exposure. Low‐dose X‐ray exposure pretreatments alone were mostly without effect as only the 1 Gy exposure significantly altered cell doubling time (Figure 2). A 75 cGy GCR exposure significantly increased cell doubling time (Figure 2). Although the lowest pretreatment (0.1 Gy) was without effect. Both the 0.5 and 1.0 Gy low‐dose X‐ray pretreatments restored the cells’ replicative ability, as the cell doubling times were significantly lower compared to the 8 Gy challenge dose alone and not different from the non‐irradiated cells (Table 4).

**TABLE 4:** *P*‐values of direct comparisons for Figure 2 (cell doubling time).

	vs no radiation	vs 75 cGy GCR
No radiation		0.0195
0.1 Gy X‐rays	0.8012	0.0779
0.5 Gy X‐rays	0.9971	0.0195
1.0 Gy X‐rays	<0.0001	0.0195
75 cGy GCR	0.0163	
75 cGy GCR + 0.1 Gy X‐rays	0.0077	0.7705
75 cGy GCR + 0.5 Gy X‐rays	0.8012	0.0583
75 cGy GCR + 1.0Gy X‐rays	0.8012	0.0008

**FIGURE 3:**
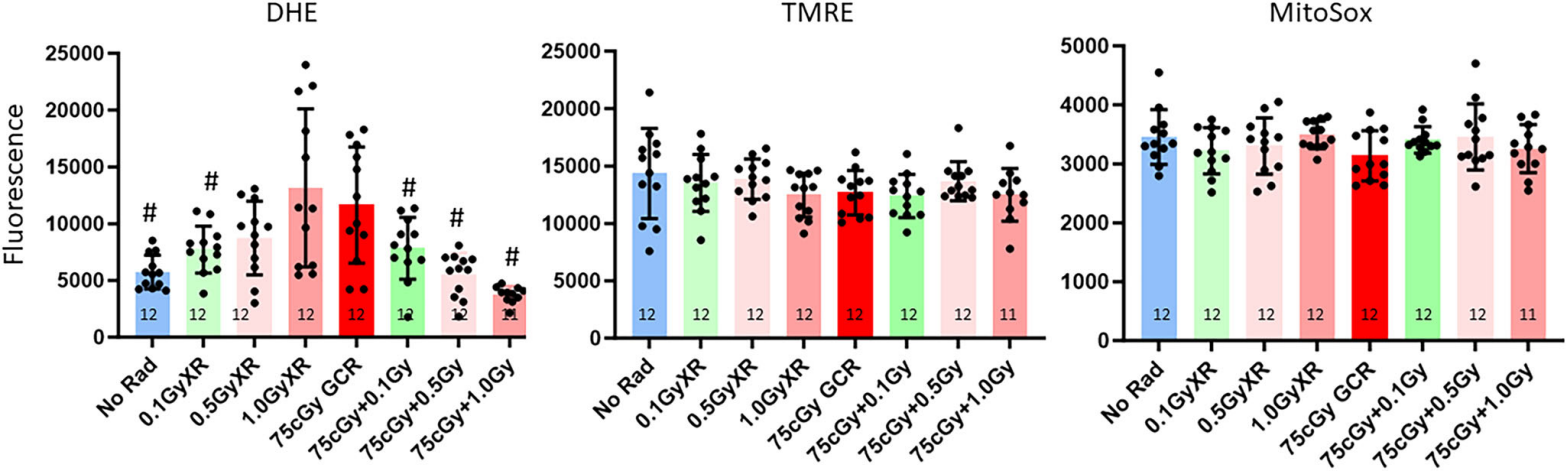
Low dose X‐rays and/or 75 cGy GCR irradiation increased cytosolic superoxide but not mitochondrial superoxide levels. Cells were exposed to control or low dose X‐rays (1.1 Gy/min) and where indicated this was followed 3 days later by the 75 cGy GCR challenge dose. Cells were maintained and analysed 7 days post‐challenge. Values shown are means ± SD with sample size indicated within each bar; #*P* < 0.05 compared to 75 cGy alone; detailed *post hoc* analysis is shown in Tables 5, 6, 7.

It is known that irradiation promotes an increase in oxidant molecules. In comparison to non‐irradiated cells, the exposure to 75 cGy GCR significantly increased cytosolic superoxide (DHE fluorescence) (Figure 3; Table 5) but not mitochondrial superoxide (MitoSox fluorescence) (Figure 3; Table 6). X‐ray pretreatments significantly lowered the GCR‐induced increases in DHE fluorescence (Table 5). Mitochondrial membrane potential, as measured with TMRE, is an indirect measure of mitochondrial viability; similar to MitoSox fluorescence, measurement of TMRE fluorescence was not influenced by either X‐ray pretreatment or the GCR challenge dose (Figure 3; Table 7).

**TABLE 5:** *P*‐values of direct comparisons for Figure 3 (DHE).

	vs no radiation	vs 75 cGy GCR
No radiation		0.0007
0.1 Gy X‐rays	0.5038	0.0364
0.5 Gy X‐rays	0.2056	0.3081
1.0 Gy X‐rays	<0.0001	0.8434
75 cGy GCR	0.0008	
75 cGy GCR + 0.1 Gy X‐rays	0.5038	0.0364
75 cGy GCR + 0.5 Gy X‐rays	0.8838	0.0005
75 cGy GCR + 1.0Gy X‐rays	0.5038	<0.0001

**TABLE 6:** *P*‐values of direct comparisons for Figure 3 (MitoSox).

	vs no radiation	vs 75 cGy GCR
No radiation		0.3142
0.1 Gy X‐rays	0.6920	0.7202
0.5 Gy X‐rays	0.8542	0.6886
1.0 Gy X‐rays	0.9905	0.2372
75 cGy GCR	0.3717	
75 cGy GCR + 0.1 Gy X‐rays	0.9905	0.3787
75 cGy GCR + 0.5 Gy X‐rays	0.9905	0.3142
75 cGy GCR + 1.0Gy X‐rays	0.7677	0.7202

**TABLE 7:** *P*‐values of direct comparisons for Figure 3 (TMRE).

	vs no radiation	vs 75 cGy GCR
No radiation		0.4546
0.1 Gy X‐rays	0.7686	0.8520
0.5 Gy X‐rays	0.7686	0.7697
1.0 Gy X‐rays	0.2739	0.8434
75 cGy GCR	0.2928	
75 cGy GCR + 0.1 Gy X‐rays	0.2655	0.9872
75 cGy GCR + 0.5 Gy X‐rays	0.7686	0.8251
75 cGy GCR + 1.0Gy X‐rays	0.2739	0.9872

**FIGURE 4:**
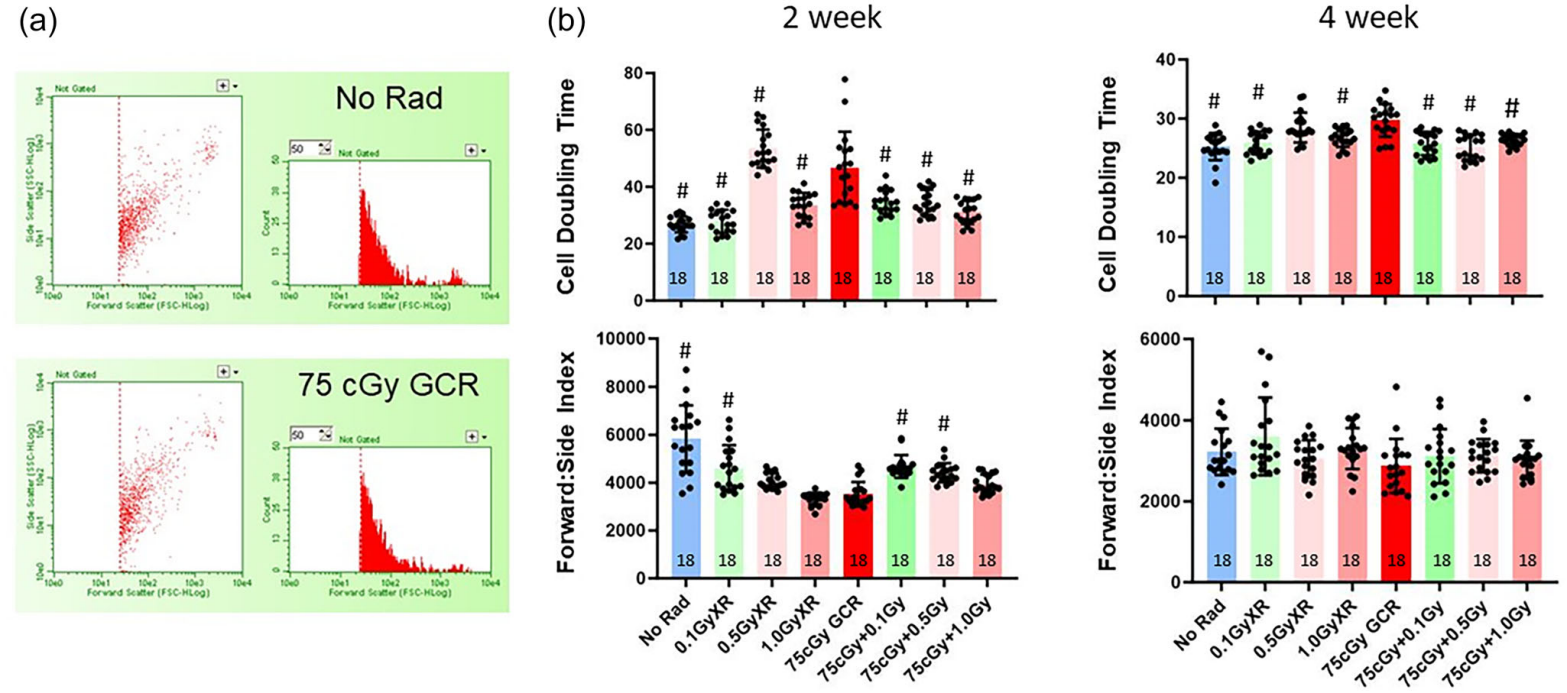
(a) Representative flow cytographs comparing Control and GCR‐exposed cells post‐treatment. (b) Impact on cell doubling time and the forward: side scatter index. H9c2 cells were exposed to combinations of low‐dose X‐rays and/or 75 cGy GCR irradiation. Cells were exposed to low dose X‐rays and where indicated this was followed 3 days later by the 75 cGy GCR challenge dose. Cells were maintained for 14 and 28 days post‐challenge. 10^5^ cells were then replated into wells of a 24‐well plate. Cells were counted after 48–72 h post‐plating. The forward: side scatter index is the product of the geometric means of each sample. Values shown are means ± SD with sample size indicated within each bar; #*P* < 0.05 compared to 75 cGy dose; detailed *post hoc* analysis is shown in Tables 8, 9, 10, 11.

Similar to analysis after 1 week, cells analysed 2 and 4 weeks post‐GCR exposure showed significantly increased cell doubling time compared to the non‐irradiated cells (Figure 4b; Tables 8 and 9). Pretreatment with low‐dose X‐rays partially restored cell proliferative ability, as cell doubling times were significantly less compared to non‐pretreated GCR exposed cells, both at the 2 and 4‐week period.

**TABLE 8:** *P*‐values of direct comparisons for Figure 4 (2‐week cell doubling time).

	vs no radiation	vs 75 cGy GCR
No radiation		<0.0001
0.1 Gy X‐rays	0.5826	<0.0001
0.5 Gy X‐rays	<0.0001	0.0013
1.0 Gy X‐rays	0.0037	<0.0001
75 cGy GCR	<0.0001	
75 cGy GCR + 0.1 Gy X‐rays	0.0008	<0.0001
75 cGy GCR + 0.5 Gy X‐rays	0.0009	<0.0001
75 cGy GCR + 1.0Gy X‐rays	0.0778	<0.0001

**TABLE 9:** *P*‐values of direct comparisons for Figure 4 (4‐week doubling time).

	vs no radiation	vs 75 cGy GCR
No radiation		<0.0001
0.1 Gy X‐rays	0.7456	<0.0001
0.5 Gy X‐rays	<0.0001	0.0799
1.0 Gy X‐rays	0.1726	<0.0001
75 cGy GCR	<0.0001	
75 cGy GCR + 0.1 Gy X‐rays	0.7456	<0.0001
75 cGy GCR + 0.5 Gy X‐rays	0.7851	<0.0001
75 cGy GCR + 1.0Gy X‐rays	0.3567	<0.0001

The flow cytometer permits determination of cell size (forward scatter) and cell complexity (side scatter) (Weiss et al., 2022). In non‐confluent cells, each measure partially reflects the cell's maturation. Shown in Figure 4a are representative cytographs of control and 75 cGy GCR irradiated cells and there is an apparent GCR‐induced decrease in the number of larger more complex cells. From these measures, we have developed a forward: side scatter index that is the product of the geometric means derived from the forward and side scatter of each sample. As shown in Figure 4b and Table 10, all the irradiation protocols significantly depressed the forward: side scatter index 2 weeks post‐GCR exposure. However, the pretreatment protocols were partially restorative as the forward: side scatter index of those cells was significantly increased compared to the GCR alone treated group. In contrast to the cell doubling times, by 4 weeks post‐GCR exposure there were no differences in the forward: side scatter index amongst the non‐irradiated and X‐ray/GCR groups (Figure 4b; Table 11).

**TABLE 10:** *P*‐values of direct comparisons for Figure 4 (2‐week forward: side index).

	vs no radiation	vs 75 cGy GCR
No radiation		<0.0001
0.1 Gy X‐rays	<0.0001	<0.0001
0.5 Gy X‐rays	<0.0001	0.0542
1.0 Gy X‐rays	<0.0001	0.4302
75 cGy GCR	<0.0001	
75 cGy GCR + 0.1 Gy X‐rays	<0.0001	<0.0001
75 cGy GCR + 0.5 Gy X‐rays	<0.0001	0.0006
75 cGy GCR + 1.0Gy X‐rays	<0.0001	0.1141

**TABLE 11:** *P*‐values of direct comparisons for Figure 4 (4‐week forward: side index).

	vs no radiation	vs 75 cGy GCR
No radiation		0.3789
0.1 Gy X‐rays	0.3706	0.0034
0.5 Gy X‐rays	0.8721	0.6281
1.0 Gy X‐rays	0.9296	0.2085
75 cGy GCR	0.4353	
75 cGy GCR + 0.1 Gy X‐rays	0.9296	0.6266
75 cGy GCR + 0.5 Gy X‐rays	0.9296	0.6266
75 cGy GCR + 1.0Gy X‐rays	0.8704	0.6281

## 4 DISCUSSION

Space travel increases solar and galactic cosmic particle radiation exposure of flight crews and this is significantly elevated once travel moves beyond LEO (Cucinotta & Durante, 2006; Cucinotta et al., 2013). Given the relatively low fluence rate of GCR exposure during space flight, it may be possible to manage these deleterious events. Our results found that pretreatment with low‐dose X‐rays enhanced cellular resiliency by inducing an adaptive response which offered a small but significant protection from a following GCR challenge.

Cellular proliferation integrates numerous pathways of functional importance for cell viability including DNA integrity as well as cellular and mitochondrial metabolism. The cell doubling time protocols examine overall cell viability and functionally parallels the clonogenic assay more commonly used (Rafehi et al., 2011). In the present study, GCR exposure significantly increased cell doubling time. That the low‐dose X‐ray pretreatment provided some protective effect suggests that compensatory pathways were activated. Although the operational effectiveness of this approach is likely to be low, potentially this approach might offer insight into cell signalling pathways that might be targeted as a countermeasure.

The use of the flow cytometer permits a measure of forward scatter and side scatter of all cells. Forward scatter is an estimator for cell size, while side scatter reflects intracellular complexity. The forward: side scatter index is the product of the geometric mean derived from the forward and side scatter of each cell and reflects cell growth dynamics. The index is analogous to the colony: area function available from ImageJ for the clonogenic assay (Guzman et al., 2014). As shown in Figure 4, GCR treatment significantly depressed the forward: side scatter index 2 weeks following the radiation exposure, but by 4 weeks no differences were observed. These data suggest that the growth pathways were transiently compromised. It is unclear if this transient suppression is the result of inactivation of endogenous proteins and their recovery or regeneration of new replacement proteins.

The forward: side scatter index is dependent upon cell size and complexity and is independent of cell number. That there were no differences at in the index at 4 weeks, but that cell doubling time was still compromised suggests the replication of DNA pathways had a longer recovery time compared to cell growth.

The underlying mechanisms for the elevated risks of GCR exposure are partially understood. GCR damages DNA, proteins and lipids. Damage occurs not only by as a direct effect but also by elevated oxidant stress promoting chronic inflammation. This occurs not only in cells directly traversed by ionized particles but also in bystander cells. On a cellular level, damaged proteins and lipids are eventually replaced. Complex pathways have evolved to manage DNA damage, and cell cycle checkpoints serve to prevent badly damaged cells from undergoing mitosis. Inside the protective buffering of the magnetosphere and our atmosphere, these pathways are mostly sufficient, but it is problematic that the higher fluency of high‐LET events produces a much higher incidence of double‐strand DNA breaks leading to mis‐repairs (Desai et al., 2005). For long‐duration missions, the cumulative impact of elevated exposure to space flight beyond LEO exceeds the capacity for repair.

Three major pathways are thought to participate in the hormetic response to radiation: (1) oxidant stress, (2) direct DNA damage, and (3) epigenetic alterations.

The diversity of oxidant molecules evidences their significant intracellular role. A dose‐dependent signalling role for the different oxidant molecules is required for normal cell growth and function, while at higher concentrations dysfunction is evident (Chen et al., 2000; Kwon et al., 2003; Valko et al., 2007). The management of oxidant molecules is responsive to their environment. Cells cultured in a very low‐radiation environment had downregulation of anti‐oxidant pathways and showed greater vulnerability to subsequent radiation exposure (Fratini et al., 2015). For high‐LET radiation events, oxidant stress is a chronic perpetrator of radiation‐induced damage (Hei et al., 2008; Suman et al., 2013; Tseng et al., 2014). Radiation‐induced oxidant stress promotes an adaptive response, in part, by increased expression of the antioxidant pathways including the Akt and Nrf2 pathways as well as antioxidant molecules such as glutathione (Liu et al., 2012; Xing et al., 2012). Bose Girigoswami & Ghosh (2005) reported that exposure to low concentrations of hydrogen peroxide generated an adaptive response that was protective from subsequent gamma radiation exposure. Not only does this point towards participation by oxidant molecules in the adaptive response to irradiation, it also suggests that oxidant molecules may serve as a mechanism for protective bystander effects (Azzam et al., 2012; Jain et al., 2011; Mladenov et al., 2018; Sisakht et al., 2020).

The functional impact of radiation‐induced alterations on epigenetic stability remains unclear. Irradiation induces both increases and decreases in DNA methylation (Belli & Indovina, 2020; Lyon et al., 2007). Increased methylation of tumour silencer promoters such as p53 may underlie tumour initiation, while global DNA hypomethylation is thought to contribute to tumour progression (Hoffmann & Schulz, 2005; Toyota & Issa, 2000). Aside from DNA methylation, radiation‐included alterations in histone structure and modification of ncRNAs have been reported (Belli & Indovina, 2020). Radiation‐induced elevated oxidant stress further promotes genomic instability (Clutton et al., 1996). Pharmacological activation of the Nrf2 pathway decreased chromosomal aberrations in response to irradiation suggesting an overlap in response to genomic instability with other aspects of the response to oxidant stress (Kim et al., 2012). These impacts on epigenetic stability are not insignificant since they directly impact on the DNA damage response, but also have persistent bystander effects (Fan et al., 2020; Koturbash et al., 2007; Koturbash et al., 2016; Sekiguchi & Matsushita, 2022).

DNA damage is a constancy for any organism, and the DNA damage response (DDR) includes genes associated with DNA repair, cell cycle and apoptosis (Jackson & Bartek, 2009). DNA damage is elevated during space flight and the incidence of high‐LET events is greatly increased. High‐LET events generate more complex DNA damage requiring longer repair periods and a greater likelihood of mis‐repairs (Desai et al., 2005; Moore et al., 2014). Activation of the p53/ATM pathways is central to the DDR across different stressors including radiation (Menendez et al., 2011; Stewart‐Ornstein et al., 2021). Zhao et al. (2015) demonstrated the effectiveness of low priming doses of radiation on the activation of p53 and attenuation of DNA damage elicited by a larger challenge dose (Zhao et al., 2015). Beyond p53 levels, p53 activation varies by cell type/metabolism suggesting a role for other DDR‐related pathways including Nrf2 and Akt (Hendrikse et al., 2000; Kim et al., 2012; Seong et al., 2001; Stewart‐Ornstein et al., 2021; Xing et al., 2012). Other studies have suggested that the radioadaptive response is stronger or more consistently observed in normal cells compared to neoplastic cells (Jiang et al., 2008; Park et al., 1999).

An adaptive response induced by low doses of irradiation may also be transmitted to bystander cells not directly irradiated. Two hours following irradiation of cultured mouse fibroblasts, their medium was transferred to non‐irradiated replicate cultures (Klammer et al., 2010). DNA repair as measured using non‐homologous end joining (D‐NHEJ) activity was significantly increased in the non‐irradiated cultures, indicating a robust adaptive response similar to the directly irradiated cells. More than just a paracrine effect, bystander effects may act at a distance, and spleen epigenetic stability was altered following irradiation of the head (Koturbash et al., 2007).

Collectively, these three pathways participate in a hormetic response to low‐dose radiation pretreatments. Any or all activation of these pathways will increase the cell's ability to manage the later challenge stress. Although in the present study we have only explored the integrated response of one cell type, the literature demonstrates the broad applicability of a radiation‐induced hormetic effect. The relative function of any one pathway will be modulated by the unique metabolism of the different cell types.

### 4.1 Conclusions

Radiation exposure from galactic cosmic radiation is a significant problem for long‐duration missions. In the present study, a cell doubling time assay was used to determine cells’ DNA integrity and cellular viability. It was found that pretreatment with low‐dose X‐rays enhanced cellular resilience by inducing an adaptive response which offered a small but significant protection against a following higher radiation challenge. Although not a practical countermeasure, these findings may serve to offer insight into cell signalling pathways activated in response to low‐dose irradiation, which might be targeted as a potential countermeasure.

## AUTHOR CONTRIBUTIONS

Siena Edwards: conceptualization, experimental design, data collection, data analysis, manuscript editing; Jessica Adams: experimental design, data collection, data analysis; Anastasia Tchernikov: data analysis, editing; John G. Edwards: experimental design, data analysis, manuscript editing. All authors have read and approved the final version of this manuscript and agree to be accountable for all aspects of the work in ensuring that questions related to the accuracy or integrity of any part of the work are appropriately investigated and resolved. All persons designated as authors qualify for authorship, and all those who qualify for authorship are listed.

## CONFLICT OF INTEREST

The authors declare no conflicts of interest.

## Data Availability

The data that support the findings of this study are available from the corresponding author upon reasonable request.
